# Accuracy and Reproducibility in Quantification of Plasma Protein Concentrations by Mass Spectrometry without the Use of Isotopic Standards

**DOI:** 10.1371/journal.pone.0140097

**Published:** 2015-10-16

**Authors:** Gertjan Kramer, Yvonne Woolerton, Jan P. van Straalen, Johannes P. C. Vissers, Nick Dekker, James I. Langridge, Robert J. Beynon, Dave Speijer, Auguste Sturk, Johannes M. F. G. Aerts

**Affiliations:** 1 Department of Medical Biochemistry, Academic Medical Centre, University of Amsterdam, Amsterdam, the Netherlands; 2 Department of Clinical Chemistry, Academic Medical Centre, University of Amsterdam, Amsterdam, the Netherlands; 3 Centre for Proteome Research, Institute of Integrative Biology, University of Liverpool, Liverpool, United Kingdom; 4 Waters Corporation, MS Technologies Centre, Manchester, United Kingdom; Leibniz-Institut für Analytische Wissenschaften - ISAS Dortmund, GERMANY

## Abstract

**Background:**

Quantitative proteomic analysis with mass spectrometry holds great promise for simultaneously quantifying proteins in various biosamples, such as human plasma. Thus far, studies addressing the reproducible measurement of endogenous protein concentrations in human plasma have focussed on targeted analyses employing isotopically labelled standards. Non-targeted proteomics, on the other hand, has been less employed to this end, even though it has been instrumental in discovery proteomics, generating large datasets in multiple fields of research.

**Results:**

Using a non-targeted mass spectrometric assay (LCMS^E^), we quantified abundant plasma proteins (43 mg/mL—40 ug/mL range) in human blood plasma specimens from 30 healthy volunteers and one blood serum sample (ProteomeXchange: PXD000347). Quantitative results were obtained by label-free mass spectrometry using a single internal standard to estimate protein concentrations. This approach resulted in quantitative results for 59 proteins (cut off ≥11 samples quantified) of which 41 proteins were quantified in all 31 samples and 23 of these with an inter-assay variability of ≤ 20%. Results for 7 apolipoproteins were compared with those obtained using isotope-labelled standards, while 12 proteins were compared to routine immunoassays. Comparison of quantitative data obtained by LCMS^E^ and immunoassays showed good to excellent correlations in relative protein abundance (r = 0.72–0.96) and comparable median concentrations for 8 out of 12 proteins tested. Plasma concentrations of 56 proteins determined by LCMS^E^ were of similar accuracy as those reported by targeted studies and 7 apolipoproteins quantified by isotope-labelled standards, when compared to reference concentrations from literature.

**Conclusions:**

This study shows that LCMS^E^ offers good quantification of relative abundance as well as reasonable estimations of concentrations of abundant plasma proteins.

## Introduction

Mass spectrometry (MS) based proteomics has various useful roles in both (clinical) research and routine diagnostics [1]. To date, clinical researchers have exploited the ability of proteomics to generate information-rich datasets of proteins, protein modifications, and potential biomarkers in various body fluids and other patient materials. This type of discovery proteomics usually consists of ‘bottom-up’ proteomics in which protein samples are digested by proteases and resulting peptides are used for identification and quantification of the constituent proteins. It routinely uses many stages of protein and peptide fractionation to generate a great number of protein identification and quantitative data and is thus inherently time consuming.

Recently, targeted proteomics-techniques have been in the spotlight in clinical proteomics, promising rapid simultaneous measurement of multiple proteins at low setup cost [2, 3]. This could alleviate bottlenecks for validating large numbers of candidate biomarkers generated in discovery proteomics in readily accessible bodily fluids like plasma. Because of this promise various studies compare plasma protein concentrations determined by targeted proteomics assays [4–11] to more routine clinical immunoassays and find correlations that range from low [5] (r = 0.43 for myeloperoxidase) to reasonable and excellent [4–11] (r = 0.63–0.99). Refinements in assay development and improved mass spectrometric techniques make the the plasma proteome of high to moderate concentration (mg/mL to ng/mL range) currently accessible without prior enrichment or fractionation [12–18]. As an example: Percey *et al*. reported the reproducible simultaneous analysis of 142 proteins with an analysis time of only ~47 minutes in non-depleted and non-enriched human plasma [15].

In contrast to targeted proteomic techniques, non-targeted proteomic approaches have so far not been tested with respect to their ability to quantify protein concentrations in clinically relevant sample matrices. This could be due to the fact that using isotope-labelled standards for each protein (as done in targeted proteomics) is impractical and costly, as illustrated by the limited number of targeted studies attempting to quantify larger protein sets using these standards. In discovery proteomics various approaches to estimate protein abundance in samples without such isotope-labelled standards have been developed. These entail either peptide or spectral counting. Examples are: EMPAI [19, 20] and APEX [21, 22], or precursor intensity based methods, such as iBAQ [23] and HI3/TOP3 peptide quantification [24, 25]. Several of these approaches have been compared in their ability to accurately determine relative or absolute protein abundance in different sample matrices [26–28]. HI3 peptide quantification, uses the sum of signal intensities of the three best ionizing peptides of any given protein and compares this to the sum of a reference protein digest spiked at a known concentration to estimate protein abundance. Protein concentrations determined by this method compare reasonably well with reference ranges in human sera [24]. Furthermore, we previously also used the HI3 peptide approach to quantify changes in the concentrations of abundant proteins in sera of Gaucher patients [29]. In this study the analysis of sera (both immuno-affinity depleted and full serum) of a small cohort of Gaucher patients showed corrections in abundant serum proteins upon treatment of patients with enzyme replacement therapy and good correlations between HI3 peptide quantitation of chitotriosidase (an important Gaucher disease biomarker) and a chitoriosidase activity assay used in routine diagnosis and disease monitoring.

In order to characterize how well HI3 peptide quantitation estimates protein concentrations in a complex sample matrix, we set out to evaluate its performance in human plasma. To do so, plasma protein concentrations are assayed by HI3 peptide quantitation and compared to those obtained using isotope-labelled standards for 7 apolipoproteins. In addition, the HI3 quantitation of plasma protein concentrations (in a cohort of 31 healthy volunteers) are compared against reference ranges and routine immunoassays conducted in parallel. The results of our investigations are presented and the potential use of non-targeted proteomics in quantitation of abundant plasma proteins is discussed.

## Materials and Methods

### Plasma Samples

Samples were obtained via the annual blood collection from healthy volunteers to prepare standard pooled plasma for diagnostic coagulation, other assays and individual plasma samples for research purposes. This is approved by the Ethical Committee at the Academic Medical Center, University of Amsterdam. Volunteers entered the blood collection event after a general call in the hospital newspaper and signed informed consent in accordance with the declaration of Helsinki. Blood samples were obtained from 31 healthy volunteers, selected from the 200 volunteers participating, individually tested for the presence of HIV, hepatitis B and C prior to the blood collection and excluded if one of the tests proved positive. This resulted in 17 males and 14 females with a median age of 46 years and a range of 22–67 years. The 30 human blood plasma samples were anonymized and had a balanced gender (16 males, 14 females) and age distribution (5–6 samples in each of the age categories 20–30, 31–40, 41–50, 51–60 and 61–70 years of age). Blood was obtained by venepuncture in 4 ml blood collection tubes (Becton Dickinson Franklin Lakes, NJ) in a final concentration of 17 IU/ml lithium-heparin. Samples were centrifuged within 15 minutes at 1780 g at 4° for 10 minutes. The plasma was then collected, divided in aliquots of 1 ml and stored at -80° within 15 minutes. The average time from collection to storage was 40 minutes. The 31st sample was a serum sample (clotting time 20 minutes followed by centrifugation at 2000 x g at 4° for 10 minutes) we processed for comparison with the results in the heparinized plasma. As results were completely comparable, the serum sample was also included in the analyses. Before use, samples were thawed at room temperature.

### Clinical Assays, reference range and assay range

Samples were processed as described above, concentrations of ceruloplasmin and serum albumin were determined nephelometrically on a BN-Prospec (Siemens, Tarrytown, NY) after immuno-complexation with their respective antisera (Siemens). Concentrations of haptoglobin, immunoglobulins alpha, gamma and mu as well as serotransferrin were determined by turbidity measurements on a Modular P800 analyzer (Roche, Basel, Switzerland) following immuno-complexation with their respective Tina-Quant antisera (Roche). After immuno-complexation with their respective antisera (Abbott, Chicago, IL), concentrations of Complement C3, C4 and apolipoproteins A1 and B-100 were determined by turbidity measurements on an ARCHITECT ci8200 (Abbott). Fibrinogen concentration was determined by measuring plasma clotting using a thrombin reagent (Siemens) on a Sysmex CA-7000 (Siemens). Reference and assay ranges are given in Table 1 for the different assays employed.

**Table 1:** Reference and assay ranges clinical assays.

Assay	Reference range (x10^6^ ng/mL)	Assay range^1^ (x10^6^ ng/mL)
Albumin	35–50	2–60
Immunoglobulin Gamma	7.0–16.0	3.0–50.0
Serotransferrin	2.0–3.6	0.10–5.2
Fibrinogen	1.5–4.0	0.3–10.0
Complement C3	0.9–1.8	0.03–3.32
Apolipoprotein A-I	1.0–2.1	0.03–3.32
Haptoglobin	0.3–2.0	0.1–5.7
Apolipoprotein B-100	0.55–1.2	0.03–2.76
Immunoglobulin Alpha	0.7–4.0	0.5–8.0
Complement C4	0.1–0.4	0.01–0.8
Ceruloplasmin	0.20–0.55	0.07–2.20
Immunoglobulin Mu	0.40–2.3	0.25–6.50

^1^Assay ranges are provided by the respective manufacturers.

### Sample preparation for LC-MS analysis

Total plasma protein concentration was assayed with a BCA-assay [30] according to the manufacturer’s protocol (Thermo). Samples were diluted tenfold in 0.1% Rapigest SF (Waters Corporation, Milford, MA), 50 mM ammonium bicarbonate and heated at 95°C for 15 min. Subsequently, plasma samples were reduced with 5 mM dithiothreitol (60°C, 30 min) and alkylated with 15 mM iodoacetamide (ambient temperature, dark, 30 min). Proteolytic digestion was performed with modified trypsin (gold grade, Promega, Madison WI) at 0.3 units/μg protein, (37°C, 20 hours) unless indicated otherwise. Following digestion, Rapigest SF was broken down by adding 1% trifluoroacetic acid (pH<2, 37°C, 45 min). Peptide solutions were centrifuged (20,000 x g, 10 min) and supernatant was collected. Prior to analyses a MASSPREP protein digestion standard (Waters Corporation, ADH1 or ENO from *Saccharomyces cerevisiae*) was added for quantitation purposes. LC-MS analyses were performed using ~ 0.21 μg of the final plasma protein digest mixtures (384 times total dilution) unless indicated otherwise.

### LC-MS analysis

Nanoscale LC separations of tryptic peptides were performed with a NanoAcquity system (Waters Corporation). Samples were loaded onto a Symmetry C18 5 μm, 2 cm x 180 μm trap column (Waters Corporation) at a flow rate of 5 μl/min prior to separation on a Bridged Ethyl Hybrid C18 1.7 μm, 25 cm x 75 μm analytical reversed phase column (Waters Corporation) by application of a 90 minute gradient from 1% acetonitrile, 0.1% formic acid to 40% acetonitrile, 0.1% formic acid at a column flow rate of 0.250 μl/min. Analysis of eluting tryptic peptides was performed using a Synapt G2 quadrupole time of flight mass spectrometer (Waters Corporation, Manchester, UK) equipped with a nanolockspray source (Waters Corporation) fitted with a pico-tip emitter (New Objective, Woburn, MA). Operated values: around 3 kV capillary voltage, cone voltage of 40 V, a source temperature of 90°C and TOF-voltage set at 7 kV. The collision gas used was argon, maintained at a constant pressure of 2.0x10^-3^ mbar in the collision cell. The lock mass, [Glu^1^]-Fibrinopeptide B, was delivered from the auxiliary pump of the NanoAcquity system at a concentration of 100 fmol/μl at 0.5 μl/min to the reference sprayer of the nanolockspray source which was sampled every 120 seconds. The data were post-acquisition lock-mass corrected using the monoisotopic mass of the doubly charged precursor of [Glu^1^]-Fibrinopeptide B. Accurate mass precursor and fragment ion LC-MS data were collected in data independent LCMS^E^ mode of acquisition [31] in the “resolution mode” of the instrument (i.e. ≥20,000 resolution at full width half maximum at 785.84 m/z). System performance was monitored by regular injections of 50 fmol ADH1 MASSPREP protein digestion standard (Waters Corporation, from *S*. *cerevisiae*). Total peptide signal intensity, retention time accuracy and chromatographic resolution were monitored and generally kept ≥70% of starting intensity, within 1% of retention time variation and within 10 seconds full width half maximum mean chromatographic peak width, respectively (system performance was assayed on a shorter 30 min gradient 0–40% acetonitrile and 0.1% formic acid).

### Data processing and protein identification

Continuum LC-MS data were processed using ProteinLynx GlobalSERVER version 2.5 (PLGS 2.5, Waters Corporation). Parameter settings: digest reagent trypsin, allow 1 ‘missed cleavage’, search tolerances automatic, typically 5 ppm for precursor and 15 ppm for product ions, fixed modification cysteine carbamidomethylation, and variable modification methionine oxidation. Protein identifications were obtained searching the human SwissProt entries of a UniProt database (release 13.2). This database was modified to include N-terminal processing of proteins using protein maturation device software [32, 33], with ADH1 and ENO1 of *S*. *cerevisiae* appended as internal standard to address technical variation and allow concentration determinations. Estimation of false-positive identification rates was done by searching a randomized version of the abovementioned human protein database generated within PLGS 2.5. Data were exported as csv-files for further, detailed analysis.

Stringent criteria were applied for quantitation, protein identifications were only considered significant if reported in 11 or more samples. Protein false positive identification rates were estimated using the criteria mentioned above and no false positives were identified in these searches. This resulted in the identification of 77 database entries (using 1498 peptide sequences). Of these 11 entries containing highly variable regions of immunoglobulins were filtered out (S1 Table).

### HI3 peptide quantitation

Label free quantitation of proteins is based on the sum of the signal intensities of the three most abundant peptides (as defined by the precursor ion area under the chromatographic peak) of a protein, (HI3(protein)) divided by the sum of the signal intensities of the three most abundant peptides of the internal standard, (HI3(standard)) times the amount in fmol of standard injected on the column [24] (Eq 1).

$$\frac{HI3(protein)}{HI3(standard)}*fmol(standard)$$ (1)

This gives an estimation of the molar amount of each protein injected on the column and PLGS 2.5 determines the molar amount (the amount in ng is determined using the molecular weight in the database) for each protein based on the ratio of its most abundant peptides determined in each individual experiment. These protein amounts determined were used for proteins that met the criteria for confident identification indicated above, to calculate the average concentration of each protein in g/L using the dilution factor of the samples. For some proteins (IgG, IgA, fibrinogen and complement C4), values of constituent polypeptide-chains were summed to obtain the protein values (see S1 Text.).

### Determination of linearity, LOD/LOQ, digestion efficiency and assay variability

To determine the amount of plasma digest to load onto a column in order for measurements to be in the linear range, a pooled plasma digest was diluted with 0.1% trifluoroaceticacid and mixed with equal amounts of internal standard (ADH1) prior to LCMS^E^ analysis and HI3 peptide quantitation. Ordinary least square linear regression was used to ascertain whether there was a linear correlation between protein amount loaded and protein amount quantified on column. From the dilution series the lowest amount (ngram) detected on column was calculated into g/L using the dilution factor (320x) of a 250 ng column load. This value, i.e. the limit of quantitation (LOQ), is reported in Table 2. As shown in S4 Fig, proteins quantified and proteins detected almost completely overlap in LCMS^E^ analysis of plasma, and as such, the lowest amount quantified on column is close to the limit of detection (LOD) as well. To test for digestion efficiency, a time series up to 24 hours of digestion is shown in S2 Fig panels a through c at 0.3 units trypsin per ug of protein. The summed HI3 peptide signals were adjusted for changes in ionisation efficiency by adjusting for the summed signals of all proteins detected. Values shown are relative to the highest summed HI3 peptide signal measured during the time series for each individual protein plotted. To ascertain which proteins change significantly in total amount quantified when using higher amounts of trypsin, a 20 hour digestion was performed with 0.15, 0.3 and 0.75 units of trypsin per ug of protein and reported in S2 Table. Assay variability was monitored by analysis of aliquots of a pooled plasma sample. The analytical variance (AV, Table 2) was calculated throughout 9 days of LCMS^E^ measurements by 10 repeated injections of a single plasma digest (n = 1). Intra-assay variation (IAV, Table 2) was determined by 6 individual digestions of aliquots of a pooled plasma sample (n = 6) and measurements during a single day. Inter-assay variability (IRV, Table 2) was determined by freezing 7 aliquots of a pooled plasma sample and thawing, digesting and measuring these over a 3 month period of normal operation of the instrument.

**Table 2:** Hi3 peptide quantitative analysis of abundant plasma proteins. (50:1 substrate:enzyme, 20 hours digestion, 0.21 ug on column injection).

**No**	**Protein name**	**median (ng/mL)**	**IRV**	**IAV**	**AV**	**LIN**	**ST**	**LOQ**	**n**	**FN**
1	Albumin	7.4 x10^7^	12	15	11	0.992	0.955	6.0 x10^3^	31	1
2	Ig gamma	4.8 x10^6^	30	17	5	0.985	0.998	8.5 x10^4^	31	2
3	Serotransferrin	2.7 x10^6^	19	19	9	0.991	0.976	2.6 x10^5^	31	3
4	Fibrinogen	2.6 x10^6^	14	15	9	0.995	0.991	7.0 x10^4^	30	4
5	Complement C3	2.3 x10^6^	17	16	7	0.987	0.974	5.6 x10^4^	31	5
6	Alpha-2-macroglobulin	1.8 x10^6^	16	11	4	0.990	0.993	5.2 x10^4^	31	6
7	Apolipoprotein A-I	1.7 x10^6^	29	14	8	0.991	0.976	2.2 x10^4^	31	7
8	Alpha-1-antitrypsin	1.6 x10^6^	14	16	15	0.985	0.964	2.7 x10^4^	31	8
9	Haptoglobin	1.3 x10^6^	26	20	9	0.993	0.991	3.2 x10^4^	31	9
10	Ig kappa chain C region	1.0 x10^6^	25	14	8	0.989	0.985	1.7 x10^4^	31	10
11	Apolipoprotein B-100	9.7 x10^5^	14	15	9	0.966	0.999	2.5 x10^5^	31	11
12	Hemopexin	9.1 x10^5^	12	12	4	0.999	0.960	2.1 x10^4^	31	12
13	Ig alpha	7.3 x10^5^	18	17	8	0.998	0.982	2.3 x10^4^	31	13
14	Complement C4	6.8 x10^5^	16	16	14	0.979	-	1.3 x10^5^	31	14
15	Fibronectin	5.0 x10^5^	-	-	32	-	-	-	22	15
16	Ig lambda chain C region	4.0 x10^5^	22	17	7	0.989	0.976	1.5 x10^4^	31	16
17	Ceruloplasmin	3.5 x10^5^	22	16	8	0.990	0.992	1.8 x10^4^	31	17
18	Inter-alpha-trypsin inhibitor hc H2	3.5 x10^5^	14	18	14	0.991	0.965	2.4 x10^4^	31	18
19	Complement factor H	2.7 x10^5^	20	20	5	0.991	0.990	3.3 x10^4^	31	19
20	Vitamin D-binding protein	2.7 x10^5^	15	15	9	0.994	0.993	3.3 x10^4^	31	20
21	Kininogen-1	2.6 x10^5^	17	14	10	0.989	0.963	3.6 x10^4^	31	21
22	Alpha-2-HS-glycoprotein	2.4 x10^5^	16	13	12	0.990	0.961	4.0 x10^3^	31	22
23	Plasminogen	2.2 x10^5^	22	20	11	0.991	0.983	4.7 x10^4^	31	23
24	Ig mu chain C region	2.2 x10^5^	26	14	17	0.993	0.997	1.4 x10^4^	31	24
25	Apolipoprotein A-II	2.0 x10^5^	27	17	6	0.995	0.988	2.4 x10^4^	31	25
26	Alpha-1-antichymotrypsin	1.8 x10^5^	16	6	6	0.989	0.961	6.0 x10^3^	31	26
27	Inter-alpha-trypsin inhibitor hc H1	1.8 x10^5^	16	-	18	0.993	-	4.4 x10^4^	31	27
28	Alpha-1-acid glycoprotein 1	1.7 x10^5^	41	16	10	0.990	0.971	6.0 x10^3^	31	28
29	Beta-2-glycoprotein 1	1.7 x10^5^	11	31	7	0.993	0.966	7.2 x10^4^	31	29
30	Inter-alpha-trypsin inhibitor hc H4	1.7 x10^5^	43	10	7	0.993	0.995	2.0 x10^4^	31	30
31	Complement factor B	1.5 x10^5^	25	52	11	0.995	0.974	2.5 x10^4^	31	31
32	Clusterin	1.5 x10^5^	10	17	10	0.990	0.958	1.7 x10^4^	31	32
33	Alpha-1B-glycoprotein	1.4 x10^5^	18	12	9	0.989	0.955	1.0 x10^4^	31	33
34	Prothrombin	1.4 x10^5^	16	23	51	0.993	0.974	1.7 x10^4^	31	34
35	Antithrombin-III	1.4 x10^5^	20	19	7	0.984	0.962	5.0 x10^3^	31	35
36	Plasma protease C1 inhibitor	1.3 x10^5^	19	18	10	0.964	0.966	1.1 x10^4^	31	36
37	Vitronectin	1.1 x10^5^	13	13	10	0.995	0.982	1.4 x10^4^	28	37
38	Apolipoprotein A-IV	1.0 x10^5^	18	16	9	0.994	0.972	9.0 x10^3^	31	38
39	C4b-binding protein alpha chain	1.0 x10^5^	18	13	35	0.996	0.997	1.5 x10^4^	31	39
40	Histidine-rich glycoprotein	9.0 x10^4^	21	13	10	0.976	0.958	8.0 x10^3^	31	40
**No**	**Protein name**	**median (ng/mL)**	**IRV**	**IAV**	**AV**	**LIN**	**ST**	**LOQ**	**n**	**FN**
41	Gelsolin	7.0 x10^4^	23	36	11	0.990	-	3.5 x10^4^	22	41
42	Heparin cofactor 2	6.6 x10^4^	17	-	100	0.991	-	1.1 x10^4^	11	42
43	Afamin	5.7 x10^4^	16	12	15	0.998	0.954	1.0 x10^5^	18	43
44	Angiotensinogen	5.7 x10^4^	19	16	5	0.989	0.992	1.1 x10^4^	30	44
45	Paraoxonase/arylesterase 1	5.1 x10^4^	22	-	22	0.982	0.999	7.0 x10^3^	23	45
46	Alpha-1-acid glycoprotein 2	5.0 x10^4^	44	15	13	0.999	0.958	4.0 x10^3^	31	46
47	Hemoglobin subunit alpha	4.1 x10^4^	40	-	7	0.991	-	3.0 x10^3^	31	47
48	Apolipoprotein C-III	3.9 x10^4^	32	27	20	-	0.978	1.4 x10^4^	31	48
49	Protein AMBP	3.9 x10^4^	26	10	16	0.825	0.949	7.0 x10^3^	22	49
50	Hemoglobin subunit beta	3.8 x10^4^	17	42	8	0.992	0.983	4.0 x10^3^	31	50
51	Pregnancy zone protein	3.6 x10^4^	64	64	75	0.959	0.998	7.0 x10^3^	15	-
52	Zinc alpha 2 glycoprotein	3.3 x10^4^	24	12	212	0.772	0.971	4.0 x10^3^	20	51
53	Apolipoprotein E	3.1 x10^4^	19	6	11	0.996	0.964	1.7 x10^4^	27	52
54	CD5 antigen like protein	2.5 x10^4^	8	7	15	-	0.997	1.3 x10^4^	19	-
55	Haptoglobin-related protein	2.3 x10^4^	24	24	21	0.968	0.995	3.0 x10^3^	16	53
56	Retinol-binding protein 4	2.2 x10^4^	20	-	27	-	-	-	14	54
57	Apolipoprotein D	2.1 x10^4^	-	-	13	-	-	-	20	55
58	Apolipoprotein C-I	1.6 x10^4^	-	-	11	-	-	-	26	56
59	Apolipoprotein C-II	1.6 x10^4^	-	23	7	-	0.922	-	19	57

**Fig 1:**
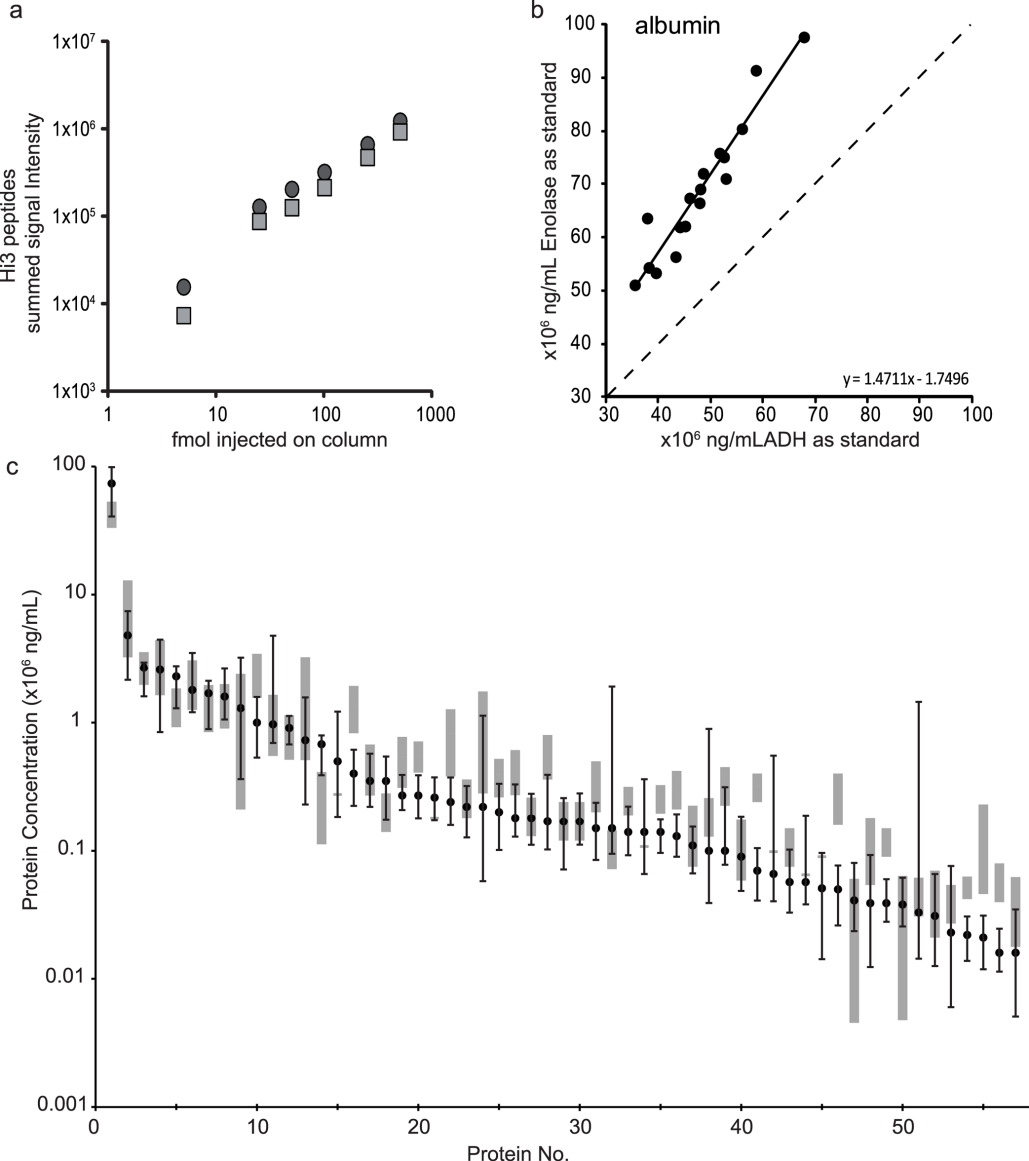
HI3 peptide quantitation with a single protein digest standard and digest standard comparison. (a) Summed signal intensity of the protein digest standard ENO1 (grey square) and ADH1 (dark grey circle) added at increasing concentrations to a plasma digest. (b) Quantitation of albumin using either ENO1 or ADH1 as the internal standard in 17 indivual samples. The regression line (solid black) and its formula, obtained by ordinary least squares linear regression, is depicted, with the dashed line representing perfect correlation. (c) 57 proteins from Table 2 for which reference ranges from literature were available, are ordered according to their median concentration determined by HI3 peptide quantitation (dark grey squares, quantified in ≥ 11 out of 31 samples). Error bars indicate the minimal and maximum value measured in the plasma samples. The reference ranges (grey boxes) are taken from Hortin *et al*. [42]. Protein no. correspond to the numbers given in Table 2.

**Median (ng/mL)**: median of protein concentrations determined from number of samples shown in column **n**. **IRV**: inter-assay variation, the coefficients of variation obtained from 7 aliquots of a pooled sample separately digested and measured over the course of 3 months of normal operation of the instrument. **IAV:** intra-assay variation, coefficient of variation obtained from 6 aliquots of a pooled sample separately digested and subsequently measured during 1 day. **AV**: analytical variability, determined from 10 replicate injections of a single digested sample throughout 9 days of measurements. **LIN:** linearity of measurements, the Pearson’s correlation coefficient shows linearity between total protein load on the analytical column and nanogram protein quantified by HI3 peptide quantitation using the ADH1 digest standard; only determined when at least 4 points were available for a protein. See also S1 Fig. **ST:** Pearson’s correlation of protein quantitation using two different digest standards (ADH1 and ENO1 from yeast). **LOQ:** limit of quantitation (ng/mL) estimated by dilution of a plasma sample in a constant background of a digest standard. **n:** number of samples (out of 31) in which the protein was quantified, **FN:** protein number on the x-axis of Fig 1c. **hc:** heavy chain.

### QconCAT production and purification

The QconCAT protein (sequence below) was produced as previously described [34] using cell lysis by sonication and purified by Ni-MAC nickel affinity column (Novagen, Merck Millipore). The QconCAT concentration was assayed with a BCA-assay [30] according to the manufacturer’s protocol (Thermo).

### Protein Sequence of QconCAT apolipoproteins

MAGREGVNDNEEGFFSAREQLGPVTQEFWDNLEKEPCVESLVSQYFQTVTDYGKDALSSVQESQVAQQARGWVTDGFSSLKEFPEVHLGQWYFIAGAAPTKESLSSYWESAKTYLPAVDEKEFGNTLEDKGFEPTLEALFGKLNILNNNYKSPELQAEAKSELEEQLTPVAEETRDYVSQFEGSALGKVLNQELREWFSETFQKVTEPISAESGEQVERTSSFALNLPTLPEVKFLLYNRLQAEAFQARLEPYADQLRLAPLAEDVRWYEIEKGVNDNEEGFFSARLAAALEHHHHHH

### LCMS^E^-QconCAT quantitation

QconCAT standard was spiked into pooled plasma samples prior to digestion as described in the *materials and methods* section at the amounts indicated (25–250 fmol/ul in a background of 250 ng/ul plasma and 50 fmol/ul ADH1 digest standard). LCMS^E^ data were acquired as described above and continuum LC-MS data were processed using ProteinLynx GlobalSERVER version 2.5. Parameter settings were as described above with additional variable modifications: ^13^C_6_-Lysine (+6.0209 amu) and ^13^C_6_-Arginine (+6.0209 amu). Data were exported as csv-files and precursor ion intensities (area under the chromatographic peak) of both endogeneous (apolipoprotein-derived) and heavy labelled (QconCAT-derived) peptides were extracted. The amount of protein in fmol was calculated as shown in (Eq 2).

$$\frac{Ion\;intensity(endogeneous\;peptide)}{Ion\;intensity\;(QconCAT\;peptide)}*fmol(QconCAT)$$ (2)

The amount obtained in fmol was subsequently used with the proteins molecular weight and sample dilution factor (320x) to calculate the plasma protein concentration in ng/mL for each peptide detected. The average plasma protein concentration of each protein was calculated by taking the average value of two peptides when available, or the single peptide value if only one of two peptides was detected. The intra-assay CV (IAV) and average protein concentration reported in S8 Table were calculated from 5 pooled plasma samples. These samples were spiked with 100 fmol/ul QconCAT (250 ng/ul plasma and 50 fmol/ul ADH1 digest standard) and measured within one day to obtain the reported values.

## Results and Discussion

### Introducing HI3 peptide quantitation and estimating its linear response range for plasma

We assayed how well quantitative results obtained by non-targeted HI3 peptide quantitation compare to those obtained by other analytical approaches such as immunoassays and proteomic approaches using isotope-labelled standards. HI3 peptide quantitation uses a reference digest standard to estimate absolute amounts of all proteins in a sample as described in Eq 1. As such, different internal digest standards should give similar responses for their HI3 peptide summed signal intensities as reported before [24]. Fig 1a shows that two different digest standards (ADH1 and ENO1) spiked into a plasma background at different concentrations give a highly similar response. Furthermore, it shows that the amount of internal digest standard used (50 fmol on column) in the HI3 peptide quantitation falls within the linear response range. The relative amounts of albumin quantified for 17 plasma samples using ADH1 or ENO1 as spiked standard are highly similar, as shown in Fig 1b. Table 2 shows the Pearson’s correlation coefficient (ST) for relative quantitation using ADH1 or ENO1 as internal standard of 50 plasma proteins (out of 59 in Table 2) is > 0.92. A small systemic difference persists, as absolute amounts estimated with ENO1 are 1.46 (SD 0.06) times higher than when ADH1 is used.

To determine that the total amount of plasma digest loaded onto the column is also in the linear response range, an increasing amount of plasma digest was injected (0.01–1.0 μg total protein). S1 Fig shows that the response was linear within this range for a subset of abundant proteins and Table 2 shows linearity (LIN) for the vast majority of proteins measured (r > 0.95). The total amount of protein loaded (~ 0.21 μg) during analysis falls within this range of linear response. In addition the limit of quantitation (LOQ) was estimated by diluting plasma in a constant background of digest standard and calculating the concentration that was still quantified in multiple injections of the dilution series (Table 2).

### Experimental variables influencing HI3 peptide quantitation: conditions of tryptic digestion

LCMS^E^ uses peptides as proxies for calculation of amounts of intact proteins; variation in digestion efficiency for proteins can have a profound impact on quantitation results while obtaining a complete digestion for *all* proteins is unlikely [4, 35–37]. To estimate which incubation time would ensure the most complete digestion for most proteins, a time series (1, 2, 3, 4, 5, 6, 7, 8, 16, 20 and 24 hrs) was performed at 0.3 units trypsin/μg protein with an MS-compatible surfactant (Rapigest SF) to aid digestion. The HI3 peptide quantitation at different time points is shown in S2 Fig panels a through c for 52 proteins that were reproducibly detected. Most proteins (S3 Fig panels a and b) show early maximisation of HI3 peptide signals within 1–2 hours of incubation with trypsin, with no or minor changes up to 24 hours of digestion. On the other hand a group of 16 proteins (S3 Fig panel c) show a definite increase of HI3 peptide signals with prolonged incubation times, indicating that these proteins require longer digestion times to reach their maximal HI3 peptide quantitation value. Amongst the proteins requiring longer digestion times 7 apolipoproteins are found. This is not surprising in light of their association in lipoprotein particles in plasma and was previously observed [4]. To test whether amounts of trypsin added significantly influences the absolute amount quantified, plasma was incubated with 0.15, 0.3 or 0.75 units per μg of total protein for 20 hours (1:100, 50 or 20 protease to protein ratio respectively). The addition of increasing amounts of trypsin does not result in significantly altered quantitation, as the majority of proteins (45 out of 52) detected show a change in quantitation of less than 1.5 fold (S2 Table). Given these results, we decided to employ a digestion time of 20 hours with 0.3 units trypsin per μg of total protein.

Here we chose for an in solution digestion protocol aided by an acid labile surfactant (Rapigest SF) to enhance protein unfolding and tryptic digestion, as a recent assessment of digestion protocols [37] showed that surfactant aided in solution protocols (among which Rapigest SF) performed similarly or better than filter aided digestion approaches [38, 39] on a (mitochondrial) protein preparation. In this study a protocol based on deoxycholate (less expensive than Rapigest SF) and phase separation rather than acid precipitation showed the best performance both in protein numbers and reproducibility. This suggests that the current approach could also benefit from this protocol at least in terms of reproducibility if not in increase of numbers of proteins quantified. Another recent report applies a digestion protocol that depletes abundant proteins in *S*. *cerevisiae* by differential digestion, called DigDeAPr [40, 41]. This could potentially increase the depth of coverage of the plasma proteome in a fashion not dissimilar from depletion of abundant plasma proteins by antibody based capture columns. This approach promises a more unbiased depletion and could certainly be useful in increasing the depth of coverage of the plasma proteome for both untargeted and targeted proteomics approaches when doing comparative studies. However, in the current study, where we also try to compare the accuracy of concentration values with regard to reference ranges it is of course counterproductive to alter protein abundancies.

### Comparing HI3 peptide quantitation to reported plasma reference ranges

To ascertain the utility of non-targeted HI3 peptide quantitation in plasma, samples collected from 31 healthy volunteers were digested and separated by reversed phase liquid chromatography before MS detection. We quantified a total of 59 proteins (631 peptides used for HI3 peptide quantitation, see S3 Table) using non-targeted LCMS^E^. Because PLGS 2.5 chooses the set of HI3 peptides to use for quantification on a per sample basis, the peptides used vary from sample to sample; for 66 database entries (59 proteins) 198 peptides would be expected if the same three peptides would be used. On average for the measurement series ~10 peptides are used per entry by PLGS 2.5 to construct HI3 quantification sets. As the quantitation is based on the ratio of summed intensities of the HI3 peptides, variation in peptides used, especially for the internal standard, can lead to variation in the absolute amount estimated by the search algorithm. The variation in 3 most intense peptides in independent samples can have a number of causes related to sample workup and analysis conditions.

To ascertain whether limiting this set of peptides manually would improve HI3 protein quantitation we manually reconstructed HI3 peptide sets for 12 proteins for which we also gathered immunoassay data (see S1 Text and S4 Table). This resulted in slight changes in median protein concentrations (S5 Table) and lower variance for two proteins as well as improved correlation with immunoassays for 4 proteins (see S1 Text and S5 Table). Because of this improvement we used the manually obtained values for these proteins in all figures and tables. However as improvement was quite limited we did not manually recalculate the values for the remaining proteins.

With regard to the 59 proteins reported in Table 2, Hortin *et al*. [42] provide reference ranges for 57 of them. Fig 1c shows the (median) plasma concentrations of these 57 proteins determined by LCMS^E^ (black circles) and their reference ranges (grey boxes). The large range for complement C4 binding protein, apolipoprotein A-IV, clusterin and heparin cofactor 2 are caused by a small number of samples (1, 3, 2 and 3 samples, respectively, see S1 Table) which have much higher concentrations than the majority of samples in which a quantitative measurement was obtained. However, as we do not have immuno-assay data for these proteins to compare to, and inter-assay variability of these proteins <30%, this could simply represent really elevated concentrations within these individuals rather than analytical error. Zinc alpha 2 glycoprotein on the other hand showed two distinct groups of samples of higher and lower concentration causing the large spread of the reported range in Fig 1c.

Comparison of median protein concentrations determined by HI3 peptides and reference ranges shows that 21 protein concentrations measured by LCMS^E^ fall within their reference ranges (S6 Table). Furthermore, median concentrations of 27 proteins are less than a factor two outside of their reference ranges. Thus, only nine (of 57) proteins fall outside their reference ranges by more than a factor two. Three reports that use targeted proteomics and stable isotope-labelled peptides quantify large number of protein concentrations in non-depleted and non-enriched human blood plasma. The protein concentrations reported in these studies are compared to the protein concentrations determined by HI3 peptide quantitation and reference ranges reported by Hortin *et al*. [42] in S6 Table. From the proteins reported, 23 are quantified both by us and all three targeted studies mentioned (S7 Table). Overall the targeted proteomics data from Kuzyk *et al*. [13] are comparable with HI3 peptide quantitation, as only 3 proteins (out of 23) were outside of their reference ranges by more than a factor of two compared to one protein for HI3 peptide quantitation. The quantitative protein data from Domanski et al. [16] and Percey et al. [15] showed higher discrepancies as 11 and 9 proteins, respectively, were outside reference ranges by more than a factor of two (S7 Table).

### Comparing HI3 peptide with stable isotope-labelled standard based quantitations

**Fig 2:**
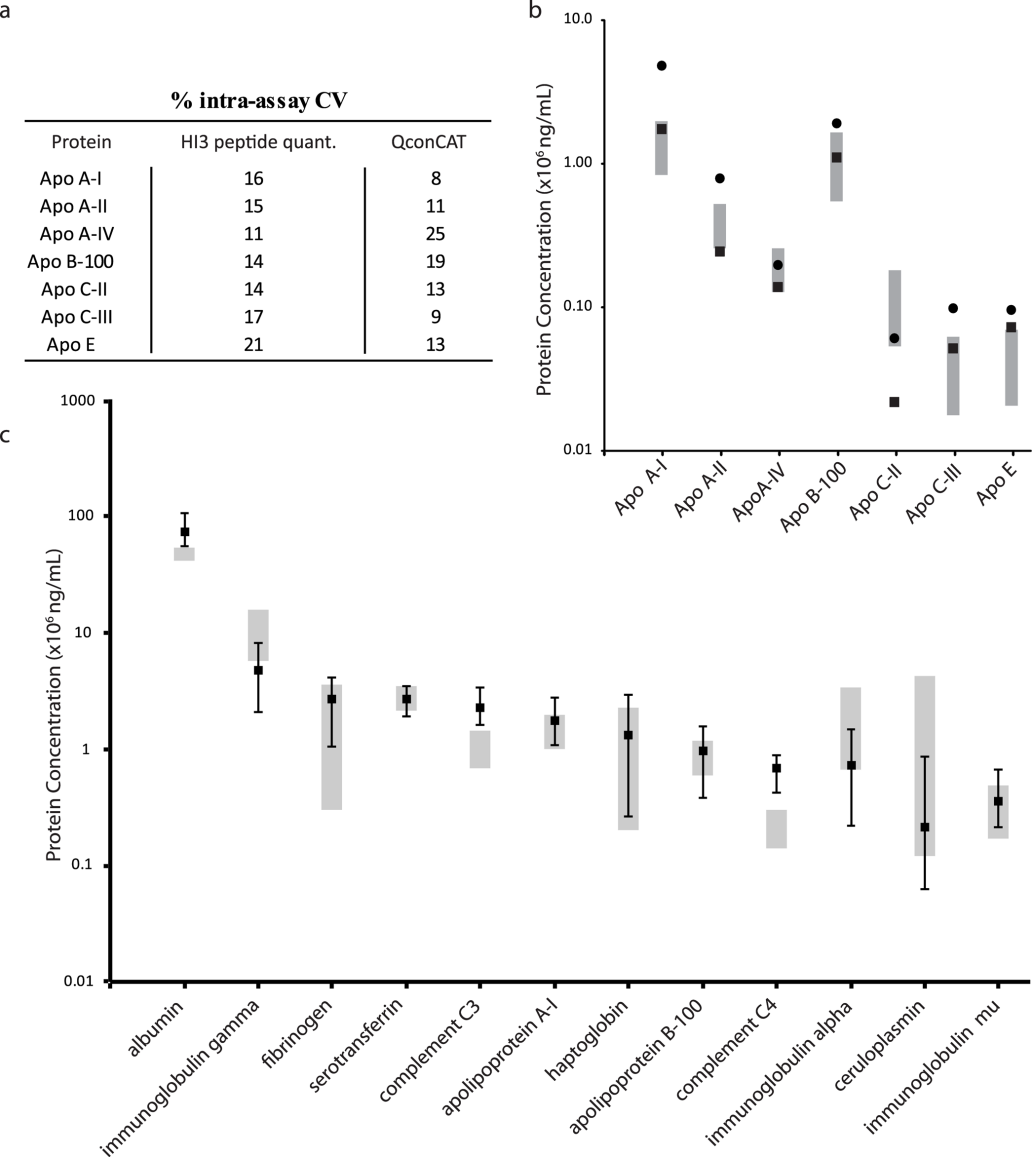
Quantitation of plasma protein concentrations by HI3, QconCAT and immunoassay. (a) Intra-assay variation of apolipoproteins by HI3 and QconCAT on a single day of measurements. (b) Protein concentration quantified by HI3 peptide quantitation (squares) or QconCAT (circles) and their reference ranges (grey boxes) in 5 pooled plasma samples. (c) Median HI3 peptide quantitation (squares) in 31 samples apart from fibrinogen (n = 30) and complement C4 (n = 29), error bars indicate the minimal and maximum value measured in the plasma samples, while grey boxes indicate ranges quantified by immunoassays in the same samples.

Next we compared concentrations quantified by HI3 peptide quantitation and stable isotope-labelled standards. For this we used an artificially constructed QconCAT protein expressed in *E*. *coli* to introduce stable isotope-labelled lysine and arginine residues. This QconCAT [43], is a concatamer of two proteotypic peptides per protein for 11 apolipoproteins. It is expressed and purified by Ni-column chromatography and was quantified by BCA-assay to ascertain the protein concentration (see materials and methods). The QconCAT was spiked into 5 pooled plasma samples within the linear response range (see S4 Fig). Following tryptic digestion and LCMS^E^ analysis, extracted ion intensities of endogenous (apolipoprotein-derived) and isotope-labelled peptides (QconCAT-derived) we could ascertain plasma protein concentrations for seven apolipoproteins (Fig 2, materials and methods). The Intra-assay variability (IAV) of quantities obtained for apolipoproteins using a QconCAT internal standard are generally lower than those obtained by HI3 quantitation (Fig 2a). Overall the apolipoprotein concentrations quantified by QconCAT are two-fold (median: 1.9, range: 1.3–3.2 fold, see S12 Table) higher than those quantified by HI3 quantitation. As digestion and measuring conditions were identical for these samples (QconCAT and HI3 quantitation was done within the same pooled plasma samples), a likely reason for this offset comes from a difference in the actual amount in fmol added of one or both standards and the value(s) used for calculation of the concentrations. As mentioned in the materials and methods, the concentration of the QconCAT was estimated by BCA-assay, while MASSPREP protein digestion standard amounts of ENO1 and ADH1 are given by the manufacturer. Another possible explanation, assuming spiking in of both standards was accurate, would be a slower release of QconCAT heavy labelled peptides compared to endogenous light peptides from the apolipoproteins which would result in a relative overestimation of concentrations. However, overall QconCATs seem to be subjected to fast and complete digestion [36]. Notwithstanding the differences in concentrations quantified, QconCAT quantitation of Apo A-IV and C-II fall within their reference ranges, whereas Apo A-II, B-100, C-III and E are less than a factor of 2 out of their reference ranges. Only the concentration of ApoA1 is more than a factor 2 out of the reference range. With HI3 quantitation, Apo A-II, Apo E are less than a factor of 2 outside their reference range, while Apo C-II is more than a factor two out of range. Overall, HI3 quantitation seems to be as close to reference ranges in plasma as the QconCAT internal standard for the apolipoproteins detected (Fig 2b).

### HI3 peptide quantitation: reliability

Reproducibility of HI3 peptide quantitation also determines the confidence with which results can be interpreted. Using the protein concentrations determined for a single pooled plasma sample, the analytical variance (AV, Table 2) was calculated throughout 9 days of LCMS^E^ measurements (median 10%, range 4–212%). Intra-assay variation (IAV, Table 2) was determined by 6 individual digestions of an aliquot of a pooled plasma sample and subsequent measurements during 1 day (median 16%, range 6–64%). Inter-assay variability (IRV, Table 2) was determined by thawing, digesting and measuring of a frozen aliquot of a pooled plasma sample over 3 months of normal operation (median 19%, range 8–64%). Zhang *et al*. [44] showed that median coefficients of variation (CVs) obtained using a single standard rather than individual standards for each individual protein can be twice as high. Median intra-assay variability of HI3 peptide quantitation is somewhat higher (Table 3) than those reported for targeted-studies employing isotope-labelled standards [12, 13], but on a par with a targeted study that did not use individual standards for each protein [14]. Overall the percentage of proteins that had an intra-assay variation <20% and <30% was somewhat lower for HI3 peptide quantitation, when compared to targeted studies that employ labelled standards (Table 3), while they were similar to a targeted study not using isotope-labelled standards.

**Table 3:** Intra-assay variation comparison of Hi3 peptide quantitation to targeted proteomics studies of plasma proteins.

Source of Data	Intra-assay CV	CV≤ 20%	CV≤ 30%
HI3 peptide quantitation data from this study.	16% (6–64%)	82%	90%
Targeted Proteomics study [12] using isotopic labels.	8% (7–12%)	100%	100%
Targeted Proteomics study [16] using isotopic labels.	6% (1–18%)	100%	100%
Targeted Proteomics study [15] using isotopic labels.	5% (1–20%)	100%	100%
Targeted Proteomics study [13] using isotopic labels.	9% (5–60%)	93%	98%
Targeted Proteomics study [14] without isotopic labels.	12% (5–40%)	76%	88%

Range of minimum to maximum value of the CV is shown between brackets.

### Comparing HI3 peptide with standard clinical immunoassay quantitation

**Fig 3:**
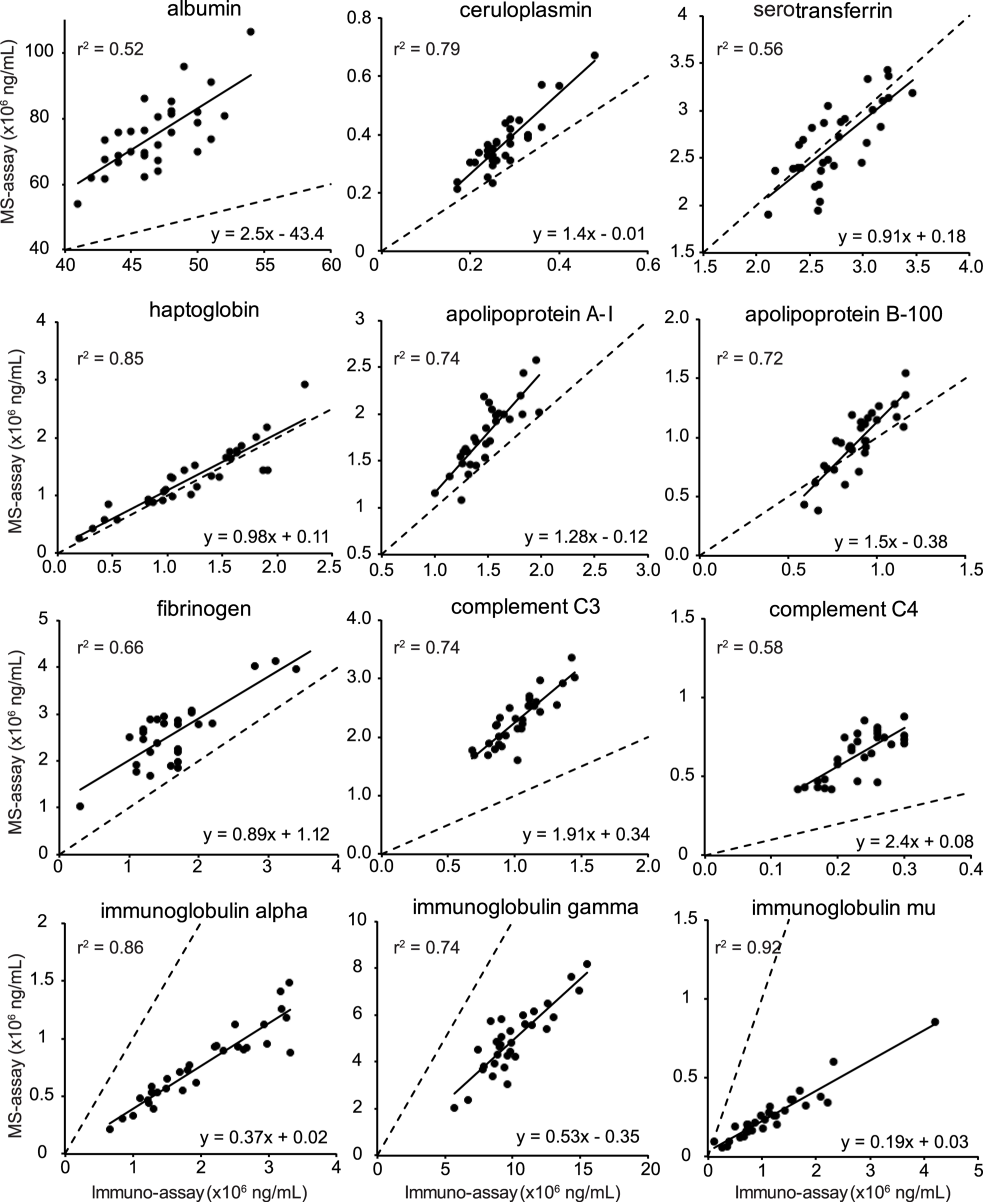
Correlation of concentrations obtained by either label free mass spectrometry or clinical immunoassay of 12 abundant plasma proteins. Each dot represents the correlation between the protein concentration (g/L) of a single sample determined by clinical immunoassays (x-axis) and label free mass spectrometry (y-axis). Regression lines (solid black) obtained by ordinary least squares linear regression and the formulas describing them are shown. The dashed line, x = y, represents perfect correlation for comparison.

We also compared the concentrations determined by HI3 peptide quantitation with those of routine clinical immunoassays in the same sample set (31 samples), which are currently the standard for plasma protein determination in clinical practice. For the 12 proteins tested, eight concentrations determined by HI3 peptide quantitation fall within the range determined by immunoassay in the same set, whereas three proteins are not more than a factor of two outside of it (Fig 2c). Only in the case of complement C4 the concentration determined by LCMS^E^ was markedly outside the immunoassay range (see Table 4 and Fig 2c). Protein concentration data obtained by LCMS^E^ were also compared to those obtained by immunoassay in *individual* samples (Fig 3). Ordinary least squares linear regression reveals linear relationships. Spearman correlation coefficients, allowing the detection of covariation in the assays, are given in Table 4. The majority of protein concentrations determined by LCMS^E^ showed good (r = 0.8–0.9) or very good correlation (r >0.9) with immunoassays. In the case of three proteins correlation is fair (>0.7). Reproducibility of immunoassays was found to be better for each of the 12 proteins examined. The median inter assay variance for HI3 peptide quantitation was 19% (12–30%) compared to 3% (1–5%) for immunoassays (Table 4).

**Table 4:** Comparison of quantification of abundant plasma proteins by HI3 peptide quantitation and clinical immunoassays.

Protein	HI3 peptide quantitation				Immunoassay			r
	median (ng/mL)	intra-assay CV^1^(%)	inter-assay CV^2^(%)	n^1^	median (ng/mL)	inter-assay CV^3^(%)	n^2^	
Albumin	7.4 x10^7^	15	12	31	4.7 x10^7^	2	31	0.72
Immunoglobulin γ	4.8 x10^6^	17	30	31	9.6 x10^6^	2	31	0.86
Transferrin	2.7 x10^6^	19	19	31	2.7 x10^6^	2	31	0.75
Fibrinogen	2.6 x10^6^	15	14	30	1.6 x10^6^	3	30	0.81*
Complement C3	2.3 x10^6^	16	17	31	1.0 x10^6^	2	31	0.86
Apolipoprotein A-I	1.7 x10^6^	14	29	31	1.5 x10^6^	4	31	0.86
Haptoglobin	1.3 x10^6^	20	26	31	1.2 x10^6^	2	31	0.92
Apolipoprotein B-100	1.0 x10^6^	15	14	31	0.9 x10^6^	3	31	0.85
Immunoglobulin α	0.7 x10^6^	17	18	31	1.8 x10^6^	1	31	0.93
Complement C4	0.7 x10^6^	16	16	29	0.2 x10^6^	3	31	0.76
Ceruloplasmin	0.4 x10^6^	16	22	31	0.3 x10^6^	5	31	0.89
Immunoglobulin μ	0.2 x10^6^	14	26	31	1.0 x10^6^	3	31	0.96

**Median (ng/mL)**: median of protein concentrations determined from number of samples shown in column **n** ^**1**^ or **n** ^**2**^.**Intra-assay CV** ^**1**^: coefficient of variation by analysis of 6 aliquots of a pooled plasma sample, digested and injected during 1 day of measurements; **Inter-assay CV** ^**2**^ by analysis of 7 aliquots of a pooled sample, digested and injected during a period of 3 months of normal operation. **n** ^**1**^, the number of samples with proteins quantified by LCMS^E^ (out of 31). Immunoassay data were obtained from the external quality control assessment scheme of the Stichting Kwaliteitscontrole Medische Laboratoria (SKML) in the Netherlands, data from September 2010. Median concentration of each analyte, inter-institute variation (**inter-assay CV** ^**3**^) and number of samples in which a protein was detected (**n** ^**2**^), are provided. r = Spearman correlation coefficient between label free MS^e^ and immunoassay values obtained for individual samples, * two outliers were removed, with outliers r = 0.54.

### Discussing the relevance of our findings for (semi)clinical settings

Our comparative study shows that using LCMS^E^ and HI3 peptide quantitation, multiple plasma proteins can be quantified in one run. We were able to quantify 59 individual plasma proteins, however only 23 proteins were quantifiable in all samples measured with an inter-assay variability ≤ 20%, (35 proteins inter-assay variability ≤ 30%). The time required for quantitation is currently two hours per sample. This multiple-protein analysis takes somewhat long compared to rapid analyses in single-protein immunoassays (~ 10 minutes), and also compared with recent targeted-proteomics studies [15–18] measuring larger numbers of proteins e.g. 142 proteins in ~47 minutes reported by Percy *et al*. [15].

We compared plasma protein concentrations determined by label free, HI3 peptide quantitation to those determined by immunoassays and targeted studies from literature using isotope-labelled standards. We also used LCMS^E^ with spiked in isotopic standards for apolipoproteins. Overall HI3 peptide quantitation determines plasma protein concentrations as well as other mass spectrometric assays employing isotopic standards in relation to reference ranges from literature. Comparison of data obtained by LCMS^E^ and immunoassay reveals a good correlation between the two. With regard to correlation with immunoassays various targeted-studies [4–9] report Spearman correlation coefficients from 0.43 to 0.99, which are for the most part comparable to those reported here.

Regarding reproducibility, the targeted studies as well as HI3 peptide quantitation have a higher variability than immunoassays used in diagnostic routine (Table 4). The low variability of immunoassays is necessary in clinical routine where individual samples are assayed against a known reference range. In comparison with targeted analysis however, variability is not much higher, within 30% for most proteins: very useful for untargeted analysis of plasma samples in discovery phases of clinical research.

In those cases where LCMS^E^ gives results differing from those reported in literature or by immunoassays it should be stressed that LCMS^E^ detects tryptic fragments of proteins. LCMS^E^ uses these peptides as proxies to calculate amounts of intact proteins. In most cases proteins quantified by LCMS^E^ tend to slightly underestimate concentrations in plasma compared to literature ranges (Fig 1c). Variation in digestion efficiency for proteins can affect quantitation [35] and incomplete digestion would generally lead to underestimation of actual protein concentration. However, as can be deduced from S2 Fig and S2 Table, the proteins underestimated are not specifically the ones increasing in quantitation with longer digestions or with more trypsin added.

On the other hand, overestimation of protein amounts (e.g. in the case of serum albumin) could be explained by the fact that some proteins in plasma may already be partially broken down into fragments from which tryptic peptides can still be generated. More generally, this phenomenon can be exacerbated under disease-conditions such as exemplified by Gaucher disease, where proteases in the circulation are abnormally high [45], and should be taken into account when doing peptide level quantification. With immunoassays, concentrations of epitopes are determined; strictly speaking, epitope concentration does not have to correlate perfectly with concentrations of intact proteins either. This is exemplified by the results of the external quality assessment of proteins in the Biorad NEQAS of March 12th 2012. In this scheme, the lowest and highest mean results of the quality control samples differed between (commercial, usually epitope-directed) antibody-based assays. Ceruloplasmin, haptoglobin, complement factors 3 and 4, IgA, IgG and IgM differed by maximum factors of 1.20, 1.35, 1.26, 1.57, 1.23. 4.87 and 1.48-fold, respectively.

## Conclusions

Our study demonstrates that LCMS^E^ allows reproducible *untargeted* quantitation of abundant plasma proteins. It gives fair to excellent correlation with immunoassays, and is achieved at low setup costs, without costly isotope-labelled standards used in targeted proteomics approaches. Reasonable variability compared to these targeted-approaches also gives confidence with regard to using this method. Furthermore, its use in investigations employing non-human model organisms with limited immunoassay availability is an attractive option. Difficulties in multiplexing immunoassays [46] combined with high setup costs mean that, despite longer analysis times, MS-based assays such as LCMS^E^ can be of interest when measuring large numbers of plasma proteins simultaneously. Although targeted approaches are more suited to validate predetermined candidate-biomarker panels in large patient cohorts, especially in plasma where targeted approaches can benefit from their larger dynamic range, the untargeted nature of LCMS^E^ and the ability to forego isotope-labelled standards still make it an attractive tool in discovery studies in clinical research settings. Use of capillary flow liquid chromatography as used in targeted studies (instead of nano-liquid chromatography) could bring down analysis times for LCMS^E^ as well, although larger sample amounts would be needed, which is not a problem for human blood plasma, but can be a limiting factor in micro-dissected disease tissues. Furthermore, addition of ion-mobility [47] as an extra dimension of separation of ions before MS^E^ detection has been shown to increase peptide and protein identification rates substantially Distler *et al*. [48] without increasing analysis time (as exemplified by quantitative results for >2500 proteins in a 90 min gradient in 200 ug Hela cell digest).

Overall, reproducibility of quantitation of the LCMS^E^ approach is acceptable for discovery studies in a (clinical) research laboratory setting [49], provided appropriate reference ranges are applied, taking into account biases of different techniques.

## Supporting Information

S1 Fig Linearity of HI3 peptide quantitation with increasing protein amount injected on column. Dilutions of a plasma proteome digest were mixed with a fixed amount of ADH1 digest standard and injected. The amount in ng quantified using HI3 peptide signals is shown for 12 selected proteins.
(PDF)

S2 Fig Digestion time series of a pooled plasma proteome sample. The digestion of a single plasma sample was followed over 24 hours. HI3 peptide summed intensities were normalized to the highest value measured in the time series for each protein
(PDF)

S3 Fig Linearity of detection of individual peptides derived from QconCAT in a plasma background. Increasing amounts of QconCAT added to a pooled plasma sample prior to digestion. Ion intensities of identified peptides are plotted against the amount (fmols) added. Formula and R^2^ obtained are depicted
(PDF)

S4 Fig Reference concentrations of proteins identified by LCMS^E^. Average concentration from Hortin et al. [42] is shown (grey dots), proteins identified by LCMS analysis are shown by blue diamonds, proteins for which a quantitative value was also determined by HI3 peptide quantitation are indicated by red squares.
(PDF)

S1 Table PGLS 2.5 Hi3 protein quantitative data. (XLSX)

S2 Table Changes in ng protein quantified on column after digestion with different amounts of trypsin. (XLSX)

S3 Table HI3 peptides in the injections as ranked by PLGS 2.5: number of injections in which a peptide was detected, variance of peak area and retention time. (XLSX)

S4 Table Manually selected HI3Peptides: number of injections detected, variance of peak area and retention time. (XLSX)

S5 Table 'Manual' versus 'automatic' peptide selection. (XLSX)

S6 Table Comparison of proteins quantified by Hi3 peptide quantitation in plasma to reference ranges and targeted studies of plasma proteins. (XLSX)

S7 Table Comparison of plasma proteins quantified by Hi3 peptide quantitation as well as three targeted proteomics studies. (XLSX)

S8 Table QconCAT quantitation of apolipoproteins in 5 pooled plasma samples. (XLSX)

S9 Table Manually recalculated plasma protein concentrations of samples and reinjected samples. (XLSX)

S10 Table Final manual HI3 and immunoassay quantitative data. (XLSX)

S11 Table Protein identifications listed per sample. (XLSX)

S12 Table Results for apolipoproteins obtained from 5 pooled plasma samples using HI3 peptide or QconCAT based quantitation compared to literature reference ranges. (XLSX)

S1 Text Supplemental Materials and Methods. (DOCX)
