# Supplementary material for: Accuracy and Reproducibility in Quantification of Plasma Protein Concentrations by Mass Spectrometry without the Use of Isotopic Standards
Source: PLoS One. 2015 Oct 16;10(10):e0140097. doi: 10.1371/journal.pone.0140097 (PMC4608811; doi:10.1371/journal.pone.0140097)
Supplement: S1 Fig — Dilutions of a plasma proteome digest were mixed with a fixed amount of ADH1 digest standard and injected. The amount in ng quantified using HI3 peptide signals is shown for 12 selected proteins. (PDF) [file pone.0140097.s001.pdf]

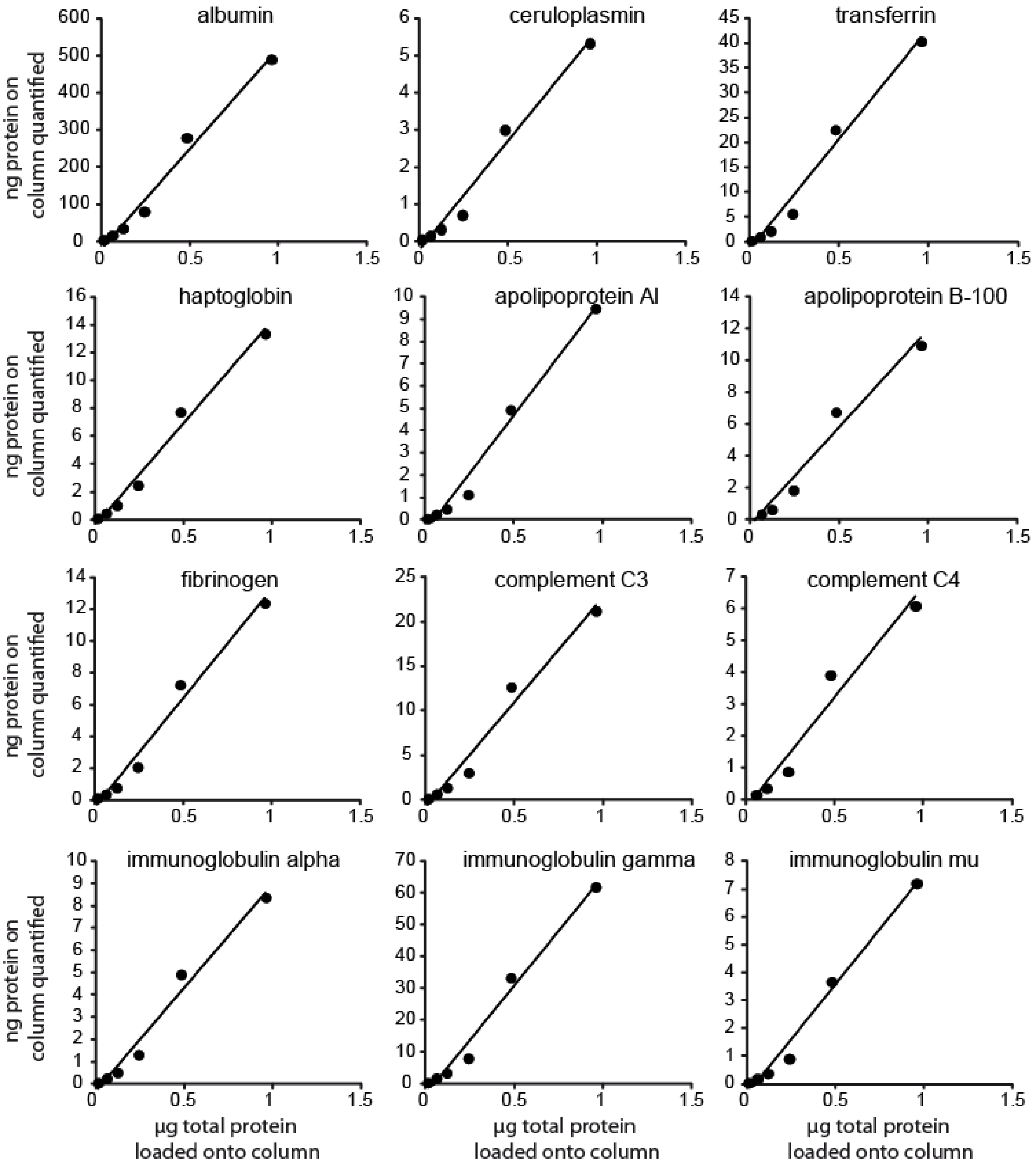

S1 Fig. Linearity of HI3 peptide quantitation with increasing protein amount injected on column. Dilutions of a plasma proteome digest were mixed with a fixed amount of ADH1 digest standard and injected. The amount in ng quantified using HI3 peptide signals is shown for 12 selected proteins.
