# Supplementary material for: Accuracy and Reproducibility in Quantification of Plasma Protein Concentrations by Mass Spectrometry without the Use of Isotopic Standards
Source: PLoS One. 2015 Oct 16;10(10):e0140097. doi: 10.1371/journal.pone.0140097 (PMC4608811; doi:10.1371/journal.pone.0140097)
Supplement: S2 Fig — The digestion of a single plasma sample was followed over 24 hours. HI3 peptide summed intensities were normalized to the highest value measured in the time series for each protein (PDF) [file pone.0140097.s002.pdf]

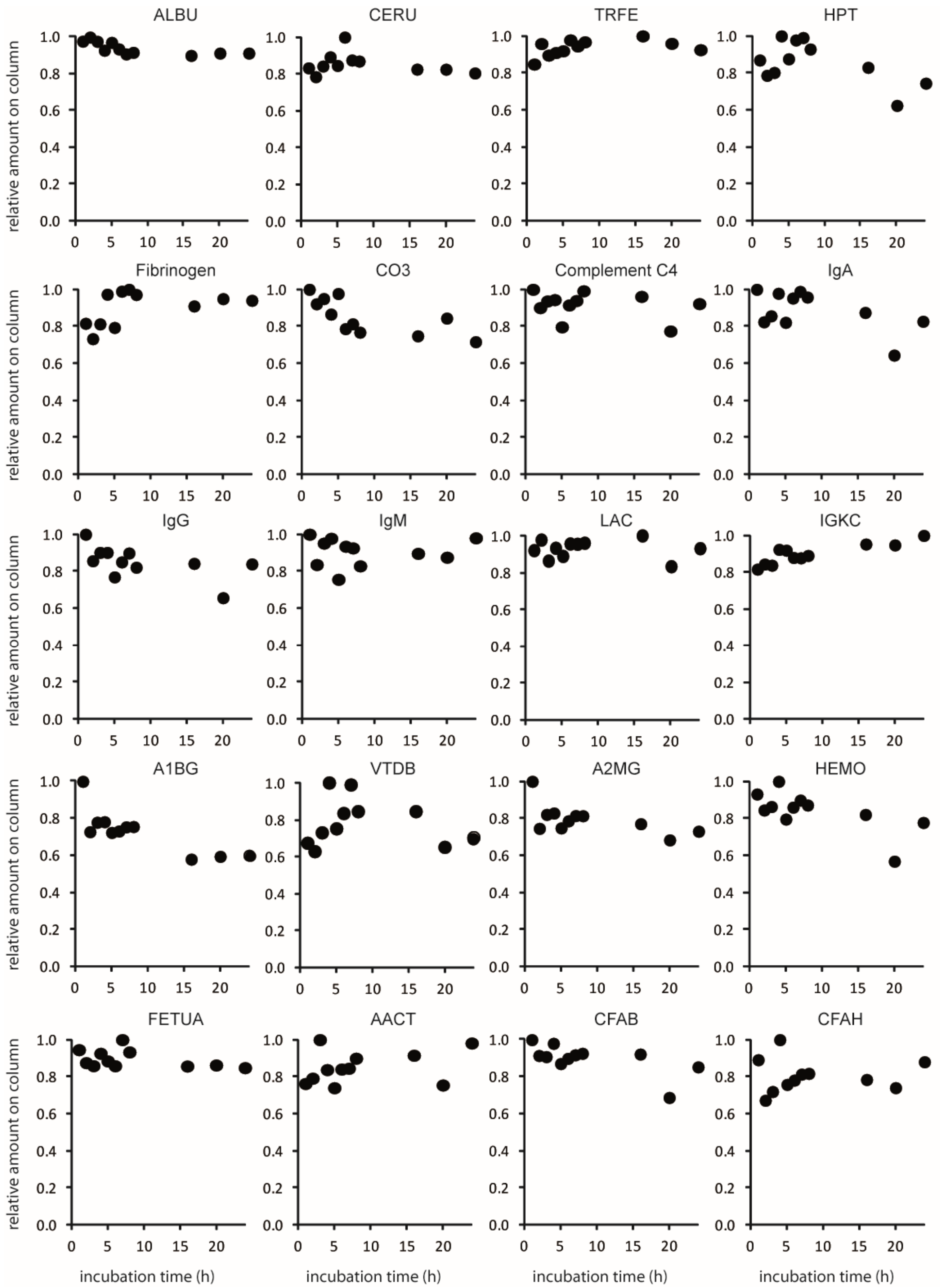

S2 Fig. a Digestion time series of a pooled plasma proteome sample. The digestion of a single plasma sample was followed over 24 hours. HI3 peptide summed intensities were normalized to the highest value measured in the time series for each protein

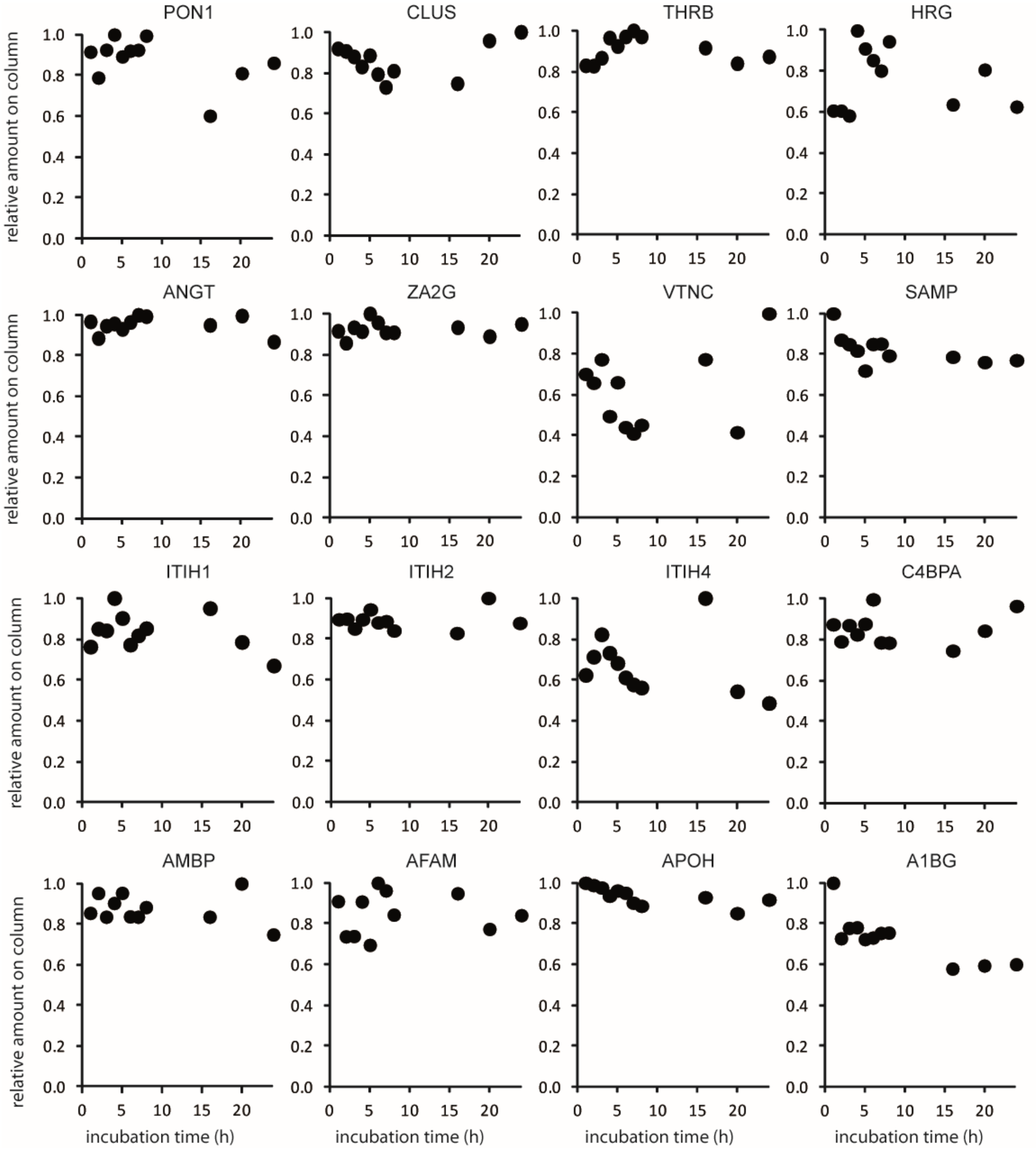

S2 Fig. b Digestion time series of a pooled plasma proteome sample. The digestion of a single plasma sample was followed over 24 hours. HI3 peptide summed intensities were normalized to the highest value measured in the time series for each protein

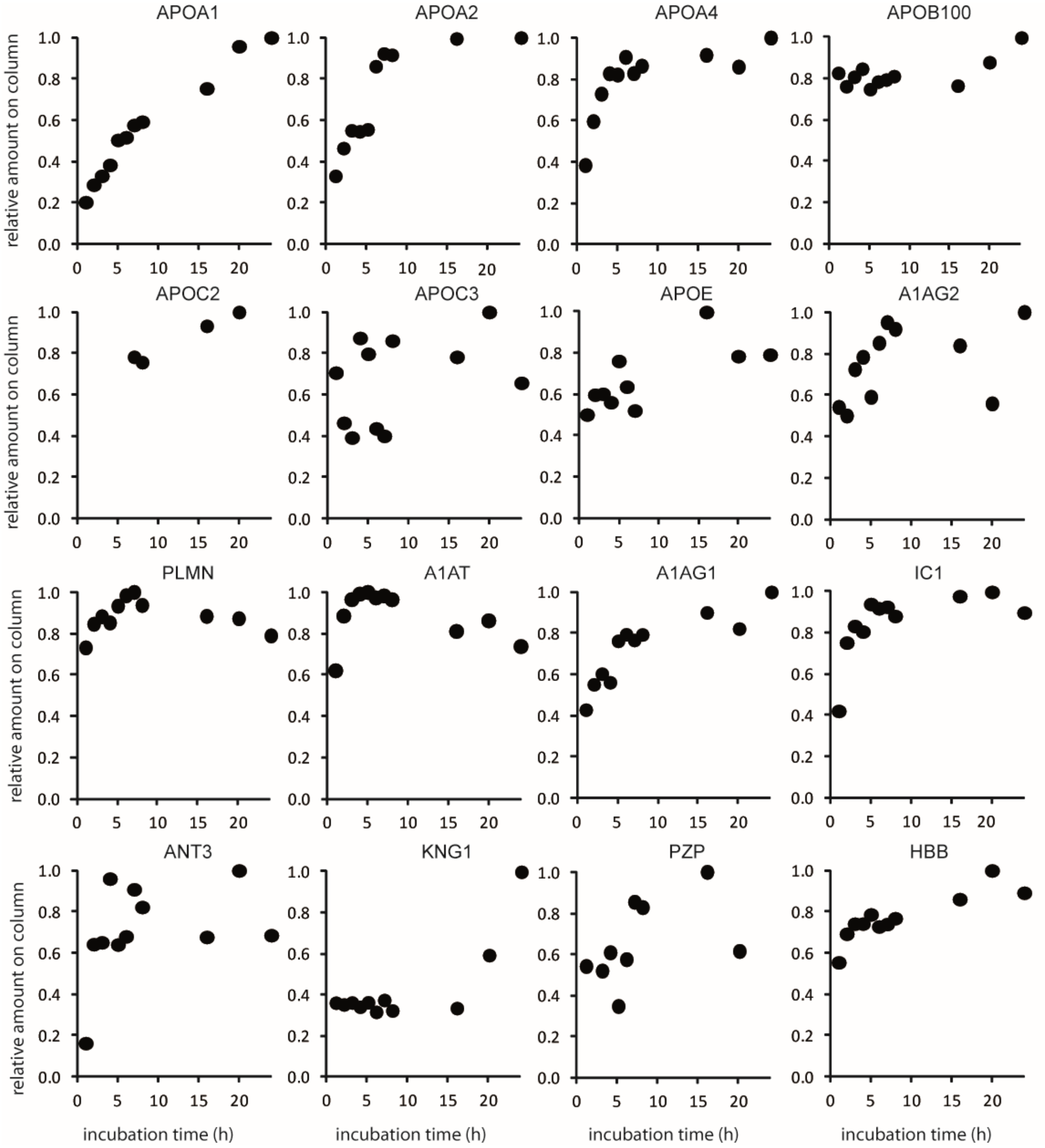

S2 Fig. c Digestion time series of a pooled plasma proteome sample. The digestion of a single plasma sample was followed over 24 hours. HI3 peptide summed intensities were normalized to the highest value measured in the time series for each protein
