# Supplementary material for: Accuracy and Reproducibility in Quantification of Plasma Protein Concentrations by Mass Spectrometry without the Use of Isotopic Standards
Source: PLoS One. 2015 Oct 16;10(10):e0140097. doi: 10.1371/journal.pone.0140097 (PMC4608811; doi:10.1371/journal.pone.0140097)
Supplement: S3 Fig — Increasing amounts of QconCAT added to a pooled plasma sample prior to digestion. Ion intensities of identified peptides are plotted against the amount (fmols) added. Formula and R2 obtained are depicted (PDF) [file pone.0140097.s003.pdf]

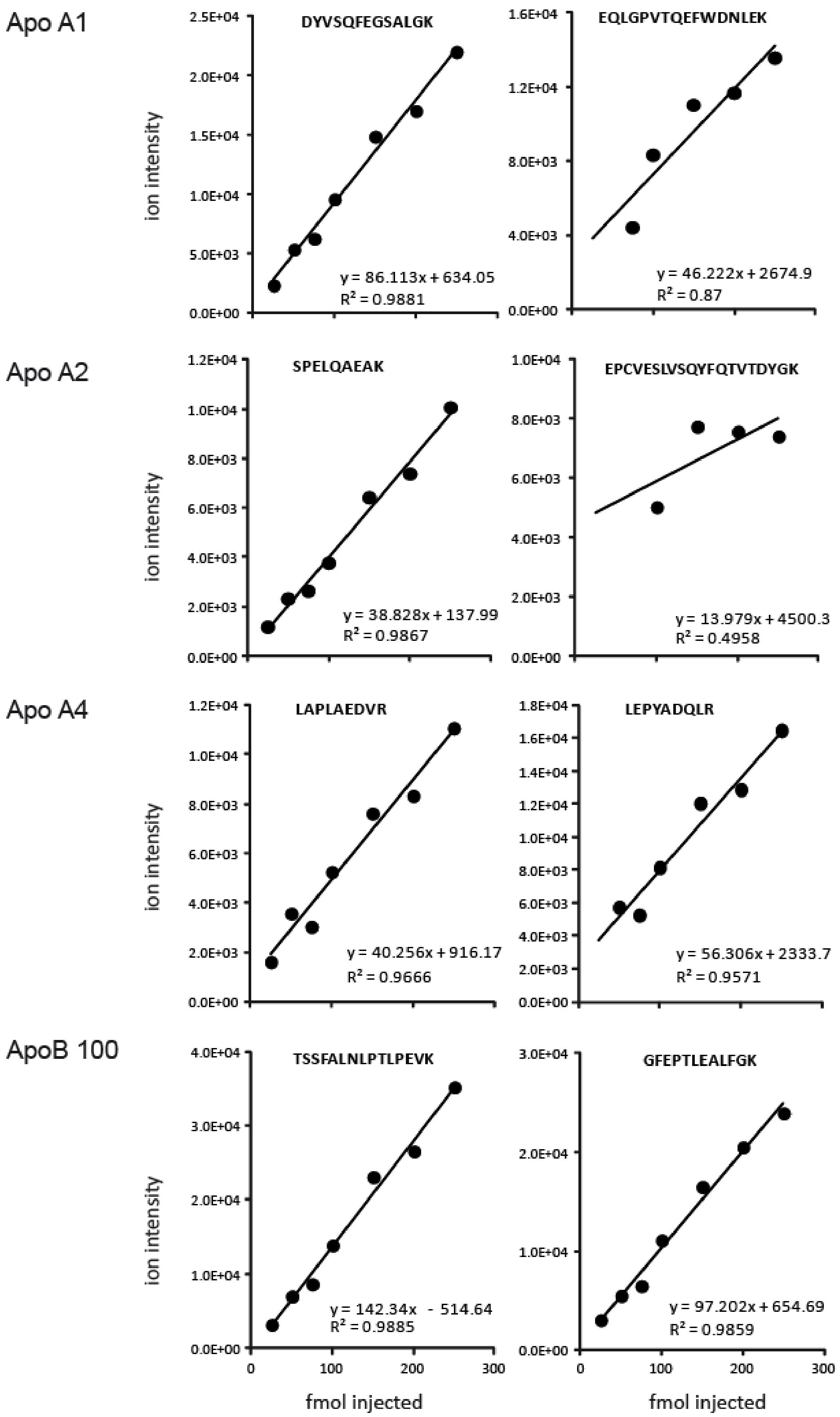

S3 Fig a. Linearity of detection of individual peptides derived from QconCAT in a plasma background. Increasing amounts of QconCAT added to a pooled plasma sample prior to digestion. Ion intensities of identified peptides are plotted against the amount (fmols) added. Formula and r^2 obtained are depicted

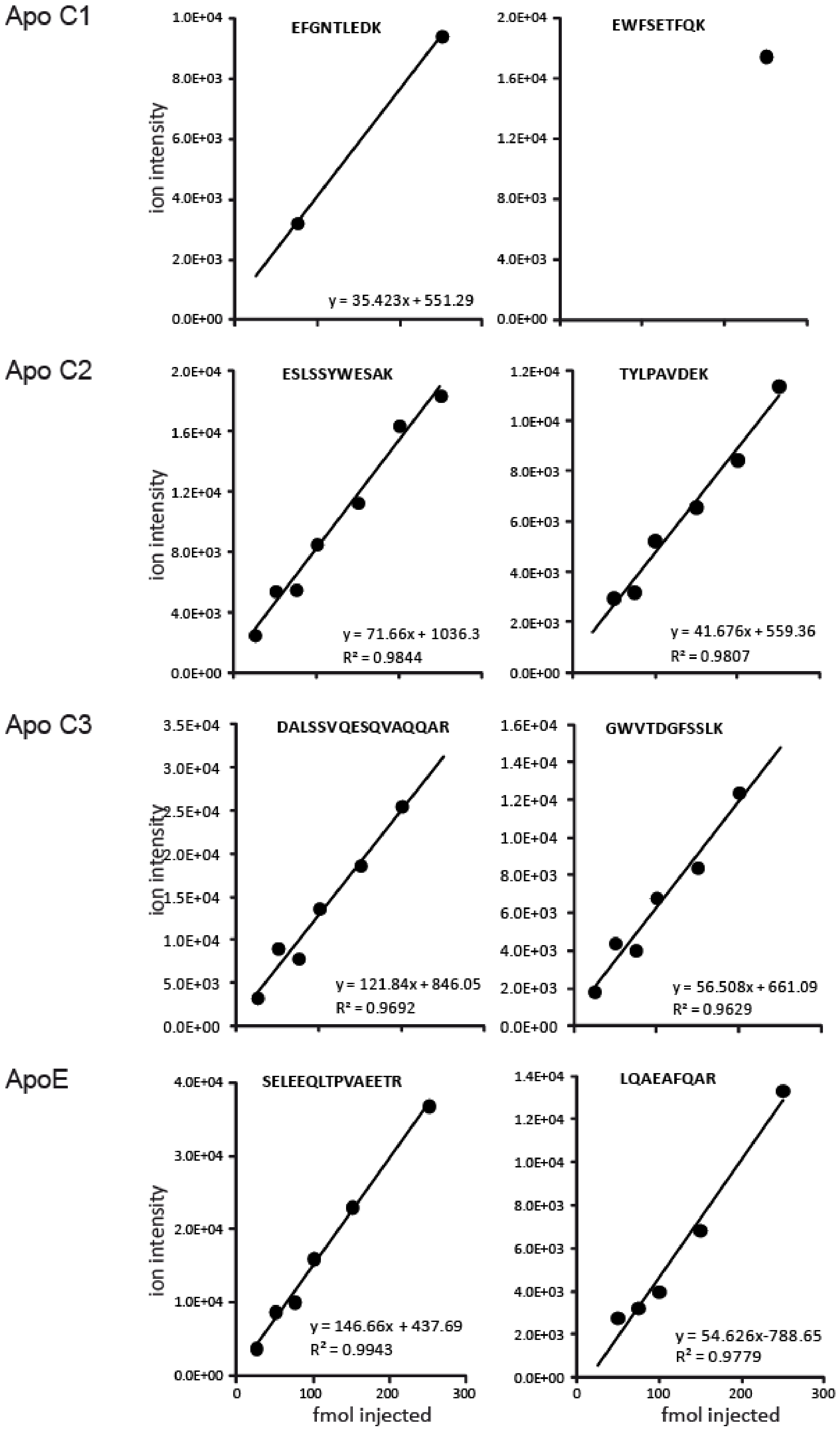

S3 Fig b. Linearity of detection of individual peptides derived from QconCAT in a plasma background. Increasing amounts of QconCAT added to a pooled plasma sample prior to digestion. Ion intensities of identified peptides are plotted against the amount (fmols) added. Formula and r^2 obtained are depicted
