# Supplementary material for: Accuracy and Reproducibility in Quantification of Plasma Protein Concentrations by Mass Spectrometry without the Use of Isotopic Standards
Source: PLoS One. 2015 Oct 16;10(10):e0140097. doi: 10.1371/journal.pone.0140097 (PMC4608811; doi:10.1371/journal.pone.0140097)
Supplement: S4 Fig — Average concentration from Hortin et al. [42] is shown (grey dots), proteins identified by LCMS analysis are shown by blue diamonds, proteins for which a quantitative value was also determined by HI3 peptide quantitation are indicated by red squares. (PDF) [file pone.0140097.s004.pdf]

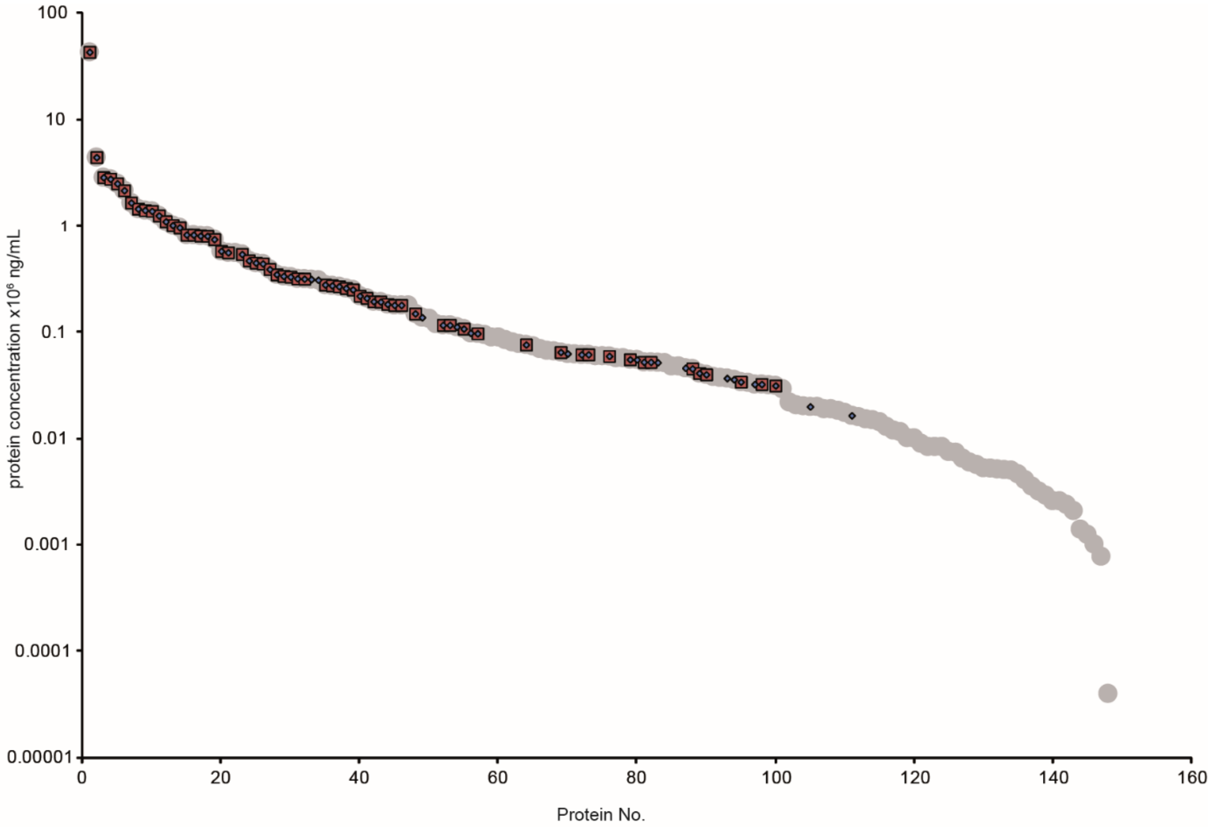

S4 Fig. Reference concentrations of proteins identified by LCMS^{E}. Average concentration from Hortin *et al.* is shown (grey dots), proteins identified by LCMS analysis are shown by blue diamonds, proteins for which a quantitative value was also determined by HI3 peptide quantitation are indicated by red squares.
