# Supplementary material for: Accuracy and Reproducibility in Quantification of Plasma Protein Concentrations by Mass Spectrometry without the Use of Isotopic Standards
Source: PLoS One. 2015 Oct 16;10(10):e0140097. doi: 10.1371/journal.pone.0140097 (PMC4608811; doi:10.1371/journal.pone.0140097)
Supplement: S1 Text — (DOCX) [file pone.0140097.s017.docx]

**Quantitation of plasma protein concentrations by mass spectrometry not using isotopic standards.**

Gertjan Kramer^1*^,Yvonne Woolerton^3^, Jan P. van Straalen^2^, Johannes P.C. Vissers^4^, Nick Dekker^1^, James I. Langridge^4^, Robert J. Beynon^3^, Dave Speijer^1^, Guus Sturk^2^ and Johannes M.F.G. Aerts^1^

**Supplemental Materials and Methods**

*Summation of polypeptide-chains to obtain protein concentrations:*

Protein amounts determined were used for proteins that met the criteria for confident identification mentioned in the materials and methods section. To calculate the average concentration of each protein in g/L the amount in ng obtained with PGLS 2.5 was multiplied by the dilution factor (386 times), used used for the samples. For some proteins, the values of different chains needed to be summed to obtain a total value for the protein. They were: Immunoglobulin α which is made up out of Immunoglobulin α 1 and 2, Immunoglobulin γ, which consists of Immunoglobulin γ 1-4, Fibrinogen, made up out of Fibrinogen α, β and γ chains, and complement C4, made up out of UniProt entries complement C4A and C4B. Values of the abovementioned proteins also needed to be summed from Hortin *et al.* (1) to obtain the reference ranges shown in Figure 1. The values of the chains were summed before average concentration, inter- and intra-assay variability or analytical variability was determined.

*Manual reanalysis of LC-MS^E^ data for 12 proteins:*

From the data generated by PLGS 2.5 as described above (because PLGS chooses the HI3 (protein) and HI3 (ADH1) peptides on a run to run basis), we noticed variation of peptides chosen throughout the entire measurement series of 31 sample injections (S8 Table). In addition, we noticed that some of the correlation graphs for the LCMS^E^ versus immunoassay quantitative data showed outliers for some proteins, as well as proteins having higher CV values. As the quantitation is based on the ratio of summed intensities of the HI3 (protein) and HI3 (ADH1) peptides, it is obvious that variation in peptides chosen for this calculation, especially for the internal standard, can lead to additional variation in the absolute amount estimated by the search algorithm. The variation in the three most intense peptides can have different causes, e.g. variation in digestion efficiency, differential ion suppression/enhancement, varying conditions on the analytical column or detection and signal interpretation mistakes.

To try and improve this, we decided to manually recalculate values for the proteins for which we also obtained immunoassay data as shown in Table 3. All peptide identification and quantitation data of the entire series were merged into a single worksheet. Here, all non-modified and fixed-modification peptides from the Table 3 proteins and the internal standard ADH1 were selected. Next, biological and technical replication rates were calculated as well as the average peptide precursor ion intensity for each sample. Peptides where then sorted, first by protein name, then by number of runs in which they are identified and finally by average intensity.

From this list 3 peptides are chosen as the HI3 (protein) value for each protein and HI3 (ADH1) for the internal standard, which preferentially have an intensity measurement in all injections, in addition to having the highest average measured intensity. Peptides with a higher average intensity but a lower number of overall detections were not considered. However, when 3 peptides were not measured in all injections, a HI3 (protein) set was calculated using alternative peptides in order to cover all injections. These manually annotated HI3 (protein) and HI3 (ADH1) sets (S9 Table) were used to estimate the amount of protein loaded in the column as described above in the following *equation*.

$$\frac{HI3(protein)}{HI3(ADH1)}*fmol\left( ADH1 \right)$$

The results of this manual re-analysis of the data differed only slightly in median concentration for the proteins (S10 Table). However, a considerable lower variance for Apolipoprotein B-100 and Complement C4 was obtained (S10 Table). Moreover, Pearson correlation coefficients for Hi3 versus Immunoassay improved by reduction of outliers for: apolipoprotein B-100, ceruloplasmin, haptoglobin and transferrin, whereas for other proteins correlation and variance did not (or hardly) change(d) (S10 Table). Following manual calculation, correlation coefficients for albumin (0.64), transferrin (0.59) and fibrinogen (0.54) remained relatively low. We re-injected the samples which showed poor correlation with immunoassays (7 samples, S14 Table), to see whether significantly different values would be obtained. This was the case for albumin and transferrin, and as such these values were used (S15 Table) to recalculate the spearman correlation coefficients (S15 Table) which improved to 0.73 (albumin) and 0.75 (transferrin), which are reported in Table 3. For Fibrinogen the values did not significantly change, the value after removing the two outliers is reported in Table 3. Because of the improvements, the manually obtained quantitative data for these 12 proteins were used in all figures and tables.
